# A Single Bout of Prolonged Sitting Augments Very Short-Term Blood Pressure Variability

**DOI:** 10.1093/ajh/hpae055

**Published:** 2024-05-04

**Authors:** Jocelyn Waghorn, Haoxuan Liu, Yanlin Wu, Sophie E Rayner, Derek S Kimmerly, Myles W O’Brien

**Affiliations:** Division of Kinesiology, Faculty of Health, School of Health and Human Performance, Dalhousie University, Halifax, Nova Scotia, Canada; Division of Kinesiology, Faculty of Health, School of Health and Human Performance, Dalhousie University, Halifax, Nova Scotia, Canada; Division of Kinesiology, Faculty of Health, School of Health and Human Performance, Dalhousie University, Halifax, Nova Scotia, Canada; Division of Kinesiology, Faculty of Health, School of Health and Human Performance, Dalhousie University, Halifax, Nova Scotia, Canada; Division of Kinesiology, Faculty of Health, School of Health and Human Performance, Dalhousie University, Halifax, Nova Scotia, Canada; Division of Geriatric Medicine (Faculty of Medicine), School of Physiotherapy (Faculty of Health), Dalhousie University, Halifax, Nova Scotia, Canada; Department of Medicine, Geriatric Medicine Research, Dalhousie University and Nova Scotia Health, Halifax, Nova Scotia, Canada; Department of Medicine, Université de Sherbrooke, Sherbrooke, Quebec, Canada; Department of Medicine, Centre de Formation Médicale Du Nouveau-Brunswick, Université de Sherbrooke, Moncton, New Brunswick, Canada

**Keywords:** beat-by-beat blood pressure, blood pressure, blood pressure dysregulation, blood pressure variability, hypertension, prolonged sitting

## Abstract

**BACKGROUND:**

More habitual time spent engaging in prolonged sedentary behaviors increases the risk of developing hypertension. Beat-by-beat systolic (SBPV) and diastolic blood pressure variability (DBPV) are more pronounced in persons with hypertension and may be an early manifestation of blood pressure dysregulation. We tested the hypothesis that a single bout of prolonged sitting augments very short-term SBPV and DBPV. The secondary aim was to explore sex differences in prolonged sitting-induced increases in SBPV and DBPV.

**METHODS:**

Thirty-three adults (22.9 ± 1.9 years; 17 females) completed a single, 3-hour bout of prolonged sitting with beat-by-beat arterial pressure determined at baseline, 1.5-hour, and 3-hour via finger photoplethysmography.

**RESULTS:**

There were no sex differences observed for baseline brachial SBP (males: 122 ± 10 mm Hg; females: 111 ± 9 mm Hg), SBPV (males: 1.87 ± 0.63 mm Hg; females: 1.51 ± 0.38 mm Hg), DBP (males: 68 ± 6 mm Hg; females: 66 ± 8 mm Hg), or DBPV (males: 1.40 ± 0.41 mm Hg; females: 1.27 ± 0.32 mm Hg) (all, *P* > 0.41). In the pooled sample, baseline SBPV (1.68 ± 0.54 mm Hg) remained unchanged after 1.5 hours (1.80 ± 0.60 mm Hg; *P* = 0.59) but increased after 3.0 hours (1.84 ± 0.52 mm Hg; *P* = 0.01). This post-sitting increase was driven by males (*P* = 0.009), with no difference observed in females (*P* = 1.00). Similarly, baseline DBPV (1.33 ± 0.36 mm Hg) was similar after 1.5 hours (1.42 ± 0.41 mm Hg; *P* = 0.72) but was increased at 3 hours (1.50 ± 0.34 mm Hg; *P* = 0.02). However, no sex differences in DBPV (all, *P* > 0.07) were observed across the time points.

**CONCLUSIONS:**

In young, normotensive adults, a single bout of prolonged sitting augmented beat-by-beat blood pressure variability, which may provide a link between uninterrupted sitting and the development of blood pressure dysregulation.

## Graphical Abstract

**Graphical Abstract:**
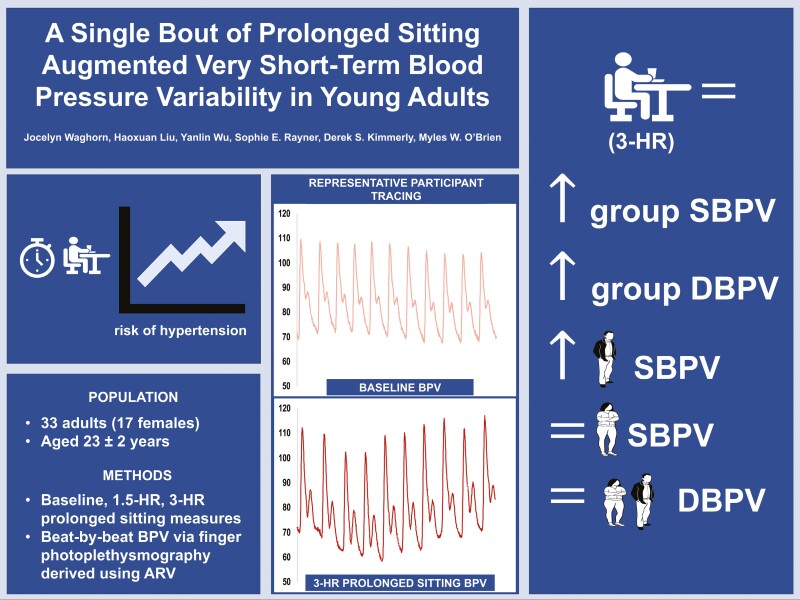

Most of our waking hours are spent engaged in sedentary behaviors,^1^ defined as any waking activity spent in a sitting, reclining, or lying posture at an energy expenditure ≤1.5 metabolic equivalents of the task.^2^ A meta-analysis of single prolonged (1–6 hours) sitting bouts demonstrated increased systolic blood pressure (SBP) and diastolic blood pressure (DBP) in young adults.^3^ Although the prevalence of hypertension increases with age, ~20% of adults aged 18–30 years exhibit hypertension, with young males often having a higher incidence of hypertension than females.^4–6^ A better understanding of the mechanistic underpinnings linking sedentariness and cardiovascular health is warranted.

Arterial blood pressure is regulated on a beat-by-beat basis via the arterial baroreflex.^7,8^ Working in concert with hemodynamic, humoral, behavioral, and environmental factors, this homeostatic response system accounts for very short-term variations in blood pressure in normotensive individuals. Although blood pressure variability may be quantified over longer time periods (e.g., 24 hours, visit-to-visit^9^), beat-by-beat systolic (SBPV) and diastolic blood pressure variability (DBPV) provides insight into the immediate physiological regulation of arterial pressure, with some research documenting that higher variability is associated with the severity of cardiovascular complications and end-organ damage in hypertensive individuals.^8–10^ Studying beat-by-beat blood pressure variability may provide unique insight into autonomic function and may be involved in the mechanistic underpinnings linking sedentary time with adverse cardiovascular health. While rates of pre-hypertension and hypertension among young-to-middle-aged adults are generally higher among males than females,^5,11,12^ resting blood pressure variability is similar between sexes.^13^ However, the impact of lifestyle factors on SBPV and DBPV among males and females is poorly understood.

It has been observed that more time spent in sedentary postures is directly related to higher resting blood pressure.^14^ In free-living conditions, our group has observed that more time spent in prolonged sedentary bouts >1 hour was associated with higher very short-term SBPV, but not DBPV, in healthy adults.^15^ However, the lack of an exposure-outcome study design prevented us from establishing a cause-and-effect relationship. Acute, single bouts of prolonged sitting (3 hours) have been utilized to study the cardiovascular consequences of sedentary postures in a laboratory setting.^16,17^ Whether SBPV and DBPV are augmented following an acute bout of sitting, and if these responses are influenced by sex are unknown.

The main objective of this study was to determine the impact of a single bout of prolonged sitting on very short-term SBPV and DBPV. Based on our free-living study,^15^ it was hypothesized that prolonged sitting would augment SBPV, but not DBPV. A secondary aim of this study was to explore whether sex differences exist in the blood pressure variability responses to prolonged sitting.

## METHODS

### Participants

Thirty-three young adults were recruited for this study (23 ± 2 years; 17 females). Participants were recruited from Dalhousie University and were eligible for inclusion if they had a body mass index (BMI) of <30 kg/m^2^, had a seated SBP <140 mm Hg and DBP <90 mm Hg, not on any blood pressure medication, and were free of any cardiovascular, metabolic, or neurological diseases. This study answers an independent, novel research question. Based on a moderate effect size (Cohen’s *f* = 0.25), correlation among repeated measure of 0.7, 90% power, an estimated sample size of *n* = 22 was needed for a repeated measure ANOVA within-between interaction (2 groups, 3 timepoints). All participants were non-obese (BMI < 30 kg/m^2^), had seated SBP: <140 mm Hg and DBP: <90 mm Hg, and 13 of 33 participants had a resting seated SBP between 120 and 139 mm Hg. No participants had been diagnosed with hypertension by a physician or were on blood pressure-lowering medications. Resting blood pressure was collected in triplicate from the left brachial artery following 10 minutes of seated rest, using an automatic vital signs monitor (Carescape v100, General Electric Healthcare). Eleven females were tested during the low estrogen phases of their menstrual cycle (*n* = 4), intrauterine device (*n* = 1), or oral contraceptive pill use (*n* = 6). All protocols and procedures followed the Declaration of Helsinki, except for registration in a database, and were approved by the Dalhousie University Health Sciences Research Ethics Board. During the initial visit, the methods and experimental design were explained to the participants verbally and in writing before written, informed consent was provided.

### Experimental design

Participants avoided foods and supplements known to have acute effects on vascular function (e.g., caffeine, chocolate, saturated fats, folic acid supplements, antioxidant supplements, and multivitamins) for 24 hours. Additionally, participants did not smoke nicotine or cannabis products or consume alcohol for 12 hours prior to the testing session. Participants were asked to abstain from moderate-vigorous activity 24 hours prior to the laboratory session. Height (to the nearest 0.1 cm) and body mass (to the nearest 0.1 kg) were collected via a calibrated stadiometer and physician’s scale, respectively, (Health-O-Meter, McCook II, USA). Body mass index was subsequently calculated.

After a standard 10-minute seated rest period and 10-minute supine rest period, participants were equipped with a three-lead, bipolar electrocardiogram, as well as brachial and finger blood pressure monitors (see details below). Once instrumented participants were positioned upright in a laboratory chair to begin the 3-hour prolonged sitting bout. Pillows placed on boxes were positioned under their feet to avoid passive muscle contractions. To minimize lower leg movements, lab personnel monitored participants throughout the session. Participants were permitted to read, do homework, or watch non-stimulating television/movies (e.g., scary movies, comedies, etc.). Blood pressure was monitored throughout the 3-hour session using finger photoplethysmography (see details below), with brachial blood pressure measurements conducted at baseline (i.e., immediately after the first 5 minutes of sitting), 1.5 hours, and 3.0 hours time points. The baseline beat-by-beat variability was determined after the participant was seated for 5 minutes. All assessments were completed in a temperature-controlled room (~21 °C). Following the prolonged sitting bout, participants were equipped with a thigh-worn activity monitor, which was worn continuously for ~1 week to assess habitual sedentary behaviors and determine activity levels.

### Blood pressure and blood pressure variability

Beat-by-beat SBP and DBP were continuously measured using finger photoplethysmography (Portapres; Finapres Medical Systems, Amsterdam, Netherlands) and were sampled at 200 Hz. Participants were equipped with two finger cuffs placed on their index or middle finger, with the cuff being transferred to the alternate finger every 30 minutes to ensure high signal integrity and participant comfort. At each of the baseline, 1.5 hours, and 3.0 hours time points, brachial SBP and DBP were measured in triplicate using an automated vital sign monitor (Carescape V100; General Electric Healthcare, Mississauga, ON, Canada) which was placed on the contralateral arm compared to the hand-fitted with the Portapres finger cuff. The average of these brachial measures was also used to calibrate the Portapres signal to the prevailing resting blood pressure. Beat-by-beat SBP and DBP values averaged over a minimum of 5 minutes (average duration: 5.5 ± 0.8 minutes) at each of the 3 time points. Heart rate was calculated from cardiac intervals derived from lead II of a standard bipolar limb lead electrocardiogram, sampled at 1000 Hz. LabChart software (Version 8.1.25; ADInstruments) was used to view the recorded signals from a PowerLab data acquisition system (PL3508 PowerLab 8/53; ADInstruments, Sydney, Australia) in real-time and offline for analysis. All data used for analyses were carefully reviewed by a trained researcher to ensure no artifacts were present.

The absolute real variability index (ARV) reflects beat-by-beat variability and is determined as the average absolute difference between successive finger blood pressure measurements.^18^ Very short-term ARV was determined for SBP and DBP at baseline, 1.5 hours, and 3.0-hour time points to determine average SBPV and DBPV across the prolonged sitting session.

### Habitual activity monitoring

To describe our sample and ensure sex comparisons were conducted between groups with comparable activity levels, each participant was equipped with an activPAL inclinometer (V3 or V4, Pal Technologies Ltd, Glasgow, UK), on the right anterior thigh, one-third of the way between the hip and knee. All participants wore the activPAL monitor 24 hours per day for up to 7 days (6.8 ± 0.4 days; range: 6–7 days). Details on how habitual physical activity and posture were determined are presented elsewhere.^15^

### Statistical analysis

All dependent variables were assessed for normality using a Shapiro–Wilk test and determined to be normally distributed (all, *P* > 0.10). Descriptive, habitual activity and baseline hemodynamic variables were compared between sexes via independent samples *t*-tests. To answer our research question, we conducted sex (males, females) by time (baseline, 1.5 hours, 3.0 hours) repeated measures analysis of variance with a covariate of SBP groupings of <120, 120–129, and 130–139 mm Hg and a Bonferroni *post hoc* testing of significant interaction effects. Additionally, bivariate correlations determined the relationships between baseline static blood pressures vs. the corresponding blood pressure variability, as well as the relationships between sitting-induced changes in average blood pressure vs. the corresponding blood pressure variability (e.g., ΔSBP vs. ΔSBPV). Statistical significance was accepted as *P* < 0.05. All statistics were conducted in SPSS V28 (IBM, NY). All data are presented as means ± standard deviation.

## RESULTS

Pooled- and sex-specific participant descriptive statistics, free-living physical activity, and free-living postural information are presented in Table 1. Participants self-reported as White (*n* = 23), Asian (*n* = 8), Arab (*n* = 1), and Black (*n* = 1). There were no differences in age, BMI, or habitual activity-related metrics (all, *P* > 0.19).

**Table 1.** Descriptive, and habitual activity outcomes for males, females, and the pooled sample

Variable	Males (*n* = 16)	Females (*n* = 17)	Total (*n* = 33)
Descriptive			
Age (y)	23 ± 2 (21–27)	23 ± 2 (20–26)	23 ± 2 (20–27)
Height (m)	1.80 ± 0.07 (1.70–1.93)	1.66 ± 0.07* (1.50–1.75)	1.73 ± 0.10 (1.50–1.93)
Weight (kg)	80.4 ± 12.7 (64.3–98.9)	64.4 ± 7.5* (56.0–81.5)	72.2 ± 13.0 (56.0–98.9)
BMI (kg/m^2^)	24.8 ± 2.9 (21.0–29.8)	23.4 ± 2.8 (20.3–29.6)	24.1 ± 2.9 (20.3–29.8)
Habitual activity			
Step count (steps/day)	10,725 ± 3,906 (7,053–20,551)	12,232 ± 2,899 (7,105–16,227)	11,501 ± 3,455 (7,053–20,551)
Standing time (min/day)	341 ± 80 (256–524)	367 ± 71 (250–512)	355 ± 75 (250–524)
Sedentary time (min/day)	519 ± 98 (295–660)	479 ± 100 (256–623)	499 ± 99 (256–660)
Prolonged sedentary time (min/day)	180 ± 85 (21–300)	178 ± 106 (24–366)	179 ± 95 (21–366)
LPA (min/day)	73 ± 28 (14–153)	66 ± 19 (5–90)	69 ± 24 (5–153)
MPA (min/day)	37 ± 17 (24–84)	44 ± 21(14–73)	41 ± 19 (14–84)
VPA (min/day)	7 ± 5 (2–21)	8 ± 10 (0–28)	7 ± 8 (0–28)
Blood pressure			
Resting SBP (mm Hg)	122 ± 10 (104–139)	111 ± 9 (96–124)	116 ± 11 (96–139)
Resting DBP (mm Hg)	68 ± 6	66 ± 8	67 ± 7
SBP <120 mm Hg	6	14	20
SBP 120–129 mm Hg	5	3	8
SBP 130–139 mm Hg	5	0	5

Data presented as means ± SD (range) or as a proportion (*n*). BMI, body mass index; HR, heart rate; LPA, light physical activity; MPA, moderate physical activity; VPA, vigorous physical activity; SBP, systolic blood pressure.

*P < 0.05 vs. males. Prolonged sedentary time was defined as >1 hour.

**Figure 1.**
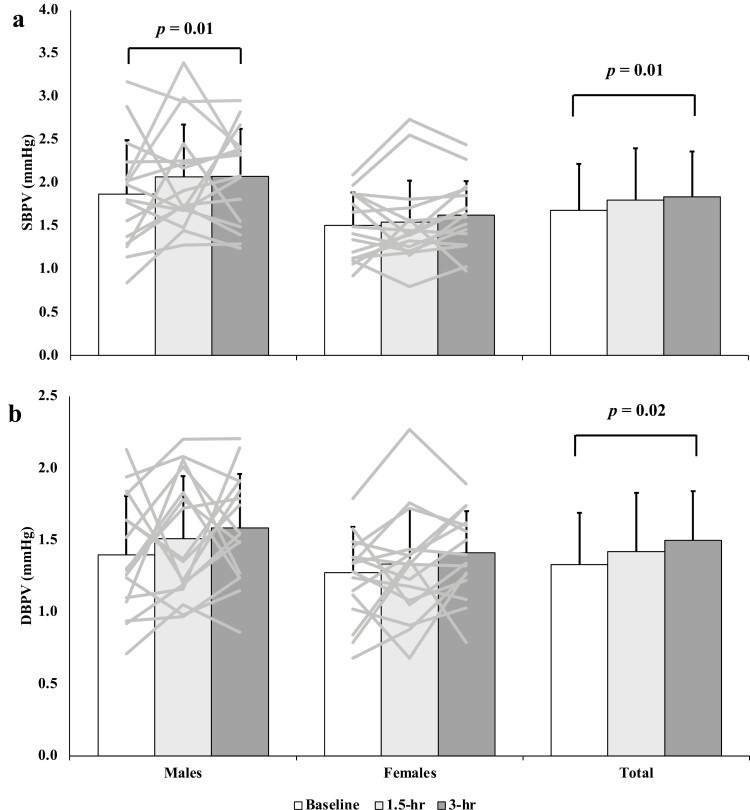
Mean and standard deviation of systolic blood pressure variability (SBPV; **a**), and diastolic blood pressure variability (DBPV; **b**), measured in mm Hg at baseline, 1.5 hours, and 3 hours for males, females, and the pooled sample. Individual participant data are represented as the gray bars shown in the male (*n* = 16) and female (*n* = 17) sections. Sex (males, females) by Time (baseline, 1.5 hours, 3.0 hours) repeated measures analysis of variance with Bonferroni *post hoc* testing was completed to assess sex and time differences. SBP category was included as a covariate. Male SBPV and DBPV were as follows: baseline (1.86 ± 0.63; 1.40 ± 0.41), 1.5 hours (2.07 ± 0.61; 1.51 ± 0.43), and 3.0 hours (2.07 ± 0.55; 1.59 ± 0.38). Female SBPV and DBPV were as follows: baseline (1.51 ± 0.38; 1.27 ± 0.32), 1.5 hours (1.55 ± 0.48; 1.34 ± 0.38), and 3.0 hours (1.63 ± 0.39; 1.41 ± 0.29). The pooled sample SBPV and DBPV were as follows: baseline (1.68 ± 0.54; 1.33 ± 0.36), 1.5 hours (1.80 ± 0.60; 1.42 ± 0.41), and 3.0 hours (1.84 ± 0.52; 1.50 ± 0.34).

In the pooled sample, SBPV was not different between baseline and the 1.5-hour sitting time point (1.68 ± 0.54 mm Hg vs. 1.80 ± 0.6 mm Hg; *P* = 0.59; Figure 1a); but was greater than baseline after 3.0 hours of sitting (1.84 ± 0.52 mm Hg; *P* = 0.01). Similarly, DBPV was unchanged between baseline and 1.5 hours of sitting (1.33 ± 0.36 mm Hg vs. 1.42 ± 0.41 mm Hg; *P* = 0.72; Figure 1b); but was increased after 3.0 hours of sitting (1.50 ± 0.34 mm Hg; *P* = 0.02).

Baseline SBPV was not higher in males vs. females (1.87 ± 0.63 mm Hg vs. 1.51 ± 0.38 mm Hg; *P* = 0.79). As well, there were no sex differences in baseline DBPV (males: 1.40 ± 0.41 mm Hg; females: 1.27 ± 0.31 mm Hg; *P* = 0.67). In males, SBPV increased from baseline to the 3-hour time point (1.87 ± 0.38 mm Hg vs. 2.07 ± 0.55 mm Hg; *P* = 0.009; Figure 1a), while in females there was no difference between baseline and 3.0 hours (1.51 ± 0.38 mm Hg vs. 1.63 ± 0.39 mm Hg; *P* = 1.00).

**Figure 2.**
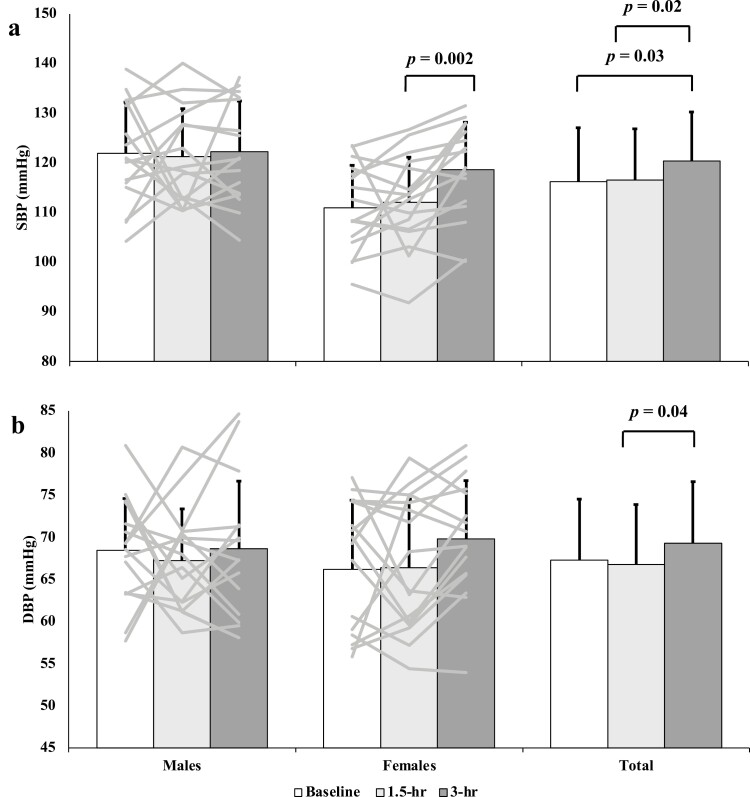
Mean and standard deviation of systolic blood pressure (SBP; **a**), and diastolic blood pressure (DBP; **b**), measured in mm Hg at baseline, 1.5 hours, and 3 hours for males, females, and the pooled sample. Individual participant data are represented as the gray bars shown in the male (*n* = 16) and female (*n* = 17) sections. Sex (males, females) by time (baseline, 1.5 hours, 3.0 hours) repeated measures analysis of variance with Bonferroni *post hoc* testing was completed to assess sex and time differences. SBP category was included as a covariate. Male SBP and DBP were as follows: baseline (122 ± 10; 68 ± 6), 1.5 hours (121 ± 10; 67 ± 6), and 3.0 hours (122 ± 10; 69 ± 8). Female SBP and DBP were as follows: baseline (111 ± 9; 66 ± 8), 1.5 hours (112 ± 9; 66 ± 8), and 3.0 hours (119 ± 10; 70 ± 7). The pooled sample SBP and DBP were as follows: baseline (116 ± 11; 67 ± 7), 1.5 hours (117 ± 10; 67 ± 7), and 3.0 hours (120 ± 10; 69 ± 7).

Brachial SBP increased from baseline to the 3.0-hour post-sitting time point (116 ± 11 mm Hg vs. 120 ± 10 mm Hg; *P* = 0.03; Figure 2a). Brachial DBP did not change from baseline to 3.0-hour post-sitting (67 ± 7 mm Hg vs. 69 ± 7 mm Hg; *P* = 0.48; Figure 2b); but did increase between the 1.5 hours and 3.0 hours (67 ± 7 mm Hg vs. 69 ± 7 mm Hg; *P* = 0.04) timepoints.

There was no difference between males and females baseline SBP (122 ± 10 mm Hg vs. 111 ± 9 mm Hg; *P* = 0.41); and also no difference in baseline DBP (males: 68 ± 6 mm Hg; females: 66 ± 8 mm Hg; *P* = 0.85).

In females, SBP was unchanged from baseline to the 3.0-hour time point (111 ± 9 mm Hg vs. 119 ± 10 mm Hg; *P* = 0.06; Figure 2a); however, there was an increase in SBP from 1.5 to 3.0 hours (*P* = 0.002). There were no differences observed in males between baseline and 3.0 hours SBP (122 ± 10 mm Hg vs. 122 ± 10 mm Hg; *P* = 0.68). Females showed no changes in DBP between 1.5 and 3.0 hours (66 ± 8 mm Hg vs. 70 ± 7 mm Hg; *P* = 0.11; Figure 2b) timepoints, or between baseline and 3.0-hour post-sitting (*P* = 0.44). There were also no changes in DBP between baseline and 3.0-hour post-sitting (69 ± 8 mm Hg; *P* = 1.00; Figure 2b), and 1.5 hours (67 ± 6 mm Hg) and 3.0 hours (*P* = 0.65) in males.

There were no relationships observed between the Δaverage pressure vs. Δpressure variability for either sex (all, *P* > 0.07). Pre-sitting average SBP and SBPV were positively related in males (*r* = 0.598, *P* = 0.01), but not in females (*P* = 0.07). Pre-sitting average DBP and DBPV were negatively related in females (*r* = −0.592, *P* = 0.01), but not in males (*P* = 0.21).

## DISCUSSION

The purpose of this study was to examine the acute impact of prolonged sitting on beat-by-beat SBPV and DBPV in young adults. Consistent with our hypothesis, SBPV was augmented by acute prolonged sitting (Figure 1a). In contrast to our hypothesis, DBPV also increased at the 3-hour timepoint (Figure 1b). SBP and DBP were also augmented by acute prolonged sitting, with both being increased by the 3-hour time point (Figure 2). These observations for blood pressure variability were driven by males, while the increased brachial SBP and DBP were primarily attributed to female participants. This study highlights the potential detriments of prolonged sitting time on the regulation of beat-by-beat blood pressure and may provide insight into the potential contributing factors by which excessive sedentariness may lead to the development of hypertension.

Single prolonged bouts of uninterrupted sitting have been well-adopted as a model to study the harmful physiological impacts of uninterrupted sedentary postures.^19,20^ Our study adds to the literature by demonstrating the negative impact of prolonged sitting on SBPV and DBPV in young adults. While pressure variability is more commonly measured over 24 hours,^10,21^ there is some early evidence that beat-by-beat SBPV and DBPV may carry important scientific and clinical relevance, with higher SBPV being associated with organ damage and cardiovascular mortality.^22^ We posit that the increase in both static brachial pressure and blood pressure variability observed after sitting 3 hours is likely an unhealthy blood pressure phenotype characterized by brief, repeated hypertensive periods that may result in blood pressure dysregulation over the life course. Our time course data substantiates that it is particularly problematic for longer periods of uninterrupted sitting, with more negative outcomes observed after 3 hours vs. 1.5 hours. Herein, partaking in longer periods of sitting without any movement (e.g., long movies, plane rides, prolonged computer work, etc.) may predispose the cardiovascular system to adverse health impacts that could explain the well-documented epidemiological link between sedentary time and hypertension.^23^

A larger blood pressure variability may be an early manifestation of blood pressure dysregulation, independent of resting blood pressure,^8^ with the beat-by-beat approach reflecting immediate cardiovascular modulation.^7^ Factors such as central artery stiffness, cardiac-specific factors (e.g., left ventricle function), and the arterial baroreflex regulate very short-term and short-term blood pressure variability.^8,24^ A single 3-hour sitting protocol observed an increased aortic pulse-wave velocity (index of aortic stiffness) among healthy young adults.^25^ The stiffening of baroreceptor-containing arteries (i.e., carotid arteries and the aorta) and the corresponding impaired distension may contribute to the augmented pressure variability. Conversely, we have documented that cardiovagal baroreflex sensitivity is not different in young males or females after 3 hours of sitting.^26^ Muscle sympathetic nerve activity is increased in seated vs. lying postures^27^ and sympathetic transduction to blood pressure is associated with increased DBPV^7^ but whether increased sympathetic vasoconstrictor activity or the transduction of this nervous activity into blood pressure responses is impacted by prolonged sitting and implicated in the augmented pressure variability with sitting warrants further research. Higher levels of sedentary time are also associated with increased left ventricle mass and left ventricle wall thickness,^28^ which may result in increased blood pressure.^29^ Altogether, the mechanisms responsible for our observations are unclear but warrant future study.

Whether sex influences the vascular responses to sitting is conflicting in studies examining endothelial function.^30,31^ The secondary purpose of this study was to determine whether sex plays a determining role in blood pressure and blood pressure variability changes across a single bout of prolonged sitting. Changes in SBPV appear to be driven by males as they were the only group to demonstrate an increase in SBPV from baseline to the 3.0-hour time point (Figure 1a). While there were no statistical differences in SBP at baseline, only females exhibited an increase in brachial SBP after 3.0 hours of sitting (Figure 2a). These findings demonstrate that males have more pronounced beat-to-beat blood pressure variation due to prolonged sitting, and although females are presented as unchanged, they still have an increase in prevailing pressure. While the physiological mechanisms responsible for this are unclear, although insignificantly different, the ~10 mm Hg lower baseline SBP and DBP among females may result in a greater room to increase vs. males whose baseline SBP and DBP were already ~120 mm Hg and ~70 mm Hg, respectively. Of note, SBP and DBP at the 3.0-hour timepoint were not different between sexes, meaning that the brachial blood pressure values for females increased to that of their male counterparts after prolonged sitting. Females tend to have a lower tolerance to orthostatic stressors than males despite comparable sympathetic neural responses to males.^32,33^ Albeit minor, prolonged sitting exhibits a gravitational stress.^34^ In a study published by Gotshall *et al*.,^35^ males exhibited a greater increase in mean arterial pressure (systolic only not presented^35^) than females after sitting for 2 hours uninterrupted. Our results conflict with such observations, which highlights the need for further research into the impact of sex and pre-sitting blood pressure on the physiologic response to prolonged sitting.

Our study may be limited by not including information pertaining to factors that impact blood pressure regulation (e.g., family disease history, smoking and alcohol consumption habits, diet, regular physical activity levels, aerobic fitness, and race). Our participants were physically active (11,501 ± 3,455 steps/day), normotensive young adults (23 ± 2 years), and our results cannot be extrapolated to older individuals with hypertension, cardiovascular disease, or persons with health complications. Studies investigating if genetic or lifestyle factors predict adverse cardiovascular responses to prolonged sitting are warranted. We were insufficiently powered to discern the impact of contraceptive use or cycle/pill phase on sitting-induced changes in BPV. Beat-by-beat SBPV and DBPV are influenced by various factors including emotions, ventilatory changes, behavior, increases in nervous system activation, etc.^36^ our exclusion of these measures limits our ability to discern the exact mechanisms responsible for our observations. A lack of a time-control (e.g., 3 hours of prolonged lying) may be considered a limitation that prevents the possibility that the elapse of 3 hours may contribute to our observations. Previous research has reported that exposure to automobile exhaust can raise blood pressure for up to 24 hours.^37,38^ As we used beat-by-beat measurements, we were unable to determine the impact of full-day sedentary time (e.g., simulated workday) on 24-hour or longer-term pressure variability. Our acute interventional design is limited in its ability to show the long-term impacts of prolonged sitting. This study is strengthened by considering both males and females and incorporation of a mid-timepoint to provide insight into the temporal responses to sitting.

## CONCLUSION

This study provides evidence that SBPV and DBPV are increased following a single 3-hour bout of prolonged sitting. This observation was primarily driven by males, but females exhibited greater increases in average SBP and DBP than males. Overall, this study provides insight into the harmful cardiovascular impacts of uninterrupted sitting in young adults and documents potential sex differences in these arterial pressure responses.

## Data Availability

Data are available from the corresponding author upon reasonable request.
